# AI ageism: a critical roadmap for studying age discrimination and exclusion in digitalized societies

**DOI:** 10.1007/s00146-022-01553-5

**Published:** 2022-10-03

**Authors:** Justyna Stypinska

**Affiliations:** 1 Freie Universität, Berlin, Germany; 2 European New School of Digital Studies, European University Viadrina, Frankfurt (Oder), Germany

**Keywords:** Ageism, Population ageing, Artificial intelligence, Digital inequalities, Age discrimination

## Abstract

In the last few years, we have witnessed a surge in scholarly interest and scientific evidence of how algorithms can produce discriminatory outcomes, especially with regard to gender and race. However, the analysis of fairness and bias in AI, important for the debate of AI for social good, has paid insufficient attention to the category of age and older people. Ageing populations have been largely neglected during the turn to digitality and AI. In this article, the concept of *AI ageism* is presented to make a theoretical contribution to how the understanding of inclusion and exclusion within the field of AI can be expanded to include the category of age. *AI ageism* can be defined as practices and ideologies operating within the field of AI, which exclude, discriminate, or neglect the interests, experiences, and needs of older population and can be manifested in five interconnected forms: (1) age biases in algorithms and datasets (technical level), (2) age stereotypes, prejudices and ideologies of actors in AI (individual level), (3) invisibility of old age in discourses on AI (discourse level), (4) discriminatory effects of use of AI technology on different age groups (group level), (5) exclusion as users of AI technology, services and products (user level). Additionally, the paper provides empirical illustrations of the way ageism operates in these five forms.

## Introduction

Over the last few years, the power of algorithms and artificial intelligence (AI) has become an issue fiercely discussed in society, media, business, and the social sciences. Artificial intelligence and machine learning (ML) systems are frequently pictured as capable of making smarter, faster, better, and presumably more neutral decisions. European Union, along with governments in Europe and around the world, see AI as the biggest promise of the twenty-first century, making positive contributions to multiple aspects of human life from improving healthcare, to climate change mitigation (European Commission 2020). At the same time, AI systems entail a number of hazards, such as opaque decision-making, gender-based, or other kinds of bias and discrimination, violation of the right to privacy, promotion of mass social engineering, and limitations to personal freedom (Rahwan 2018; Tufekci 2014). The scholars of ethical AI express concern about the way those technologies show hidden biases resulting in exclusion and discrimination of members of marginalized groups (Eubanks 2018; Mittelstadt et al. 2016) and can pose a threat to fundamental human rights and social justice (Aizenberg and van den Hoven 2020; Cruz 2020).

Particularly, the way sexism and racism operate in AI has attracted significant attention from prominent scholars. Studies have shown how face recognition systems work poorly for women with dark skin (Buolamwini and Gebru 2018) and that word embeddings—a framework used for text analysis in machine learning and neural language processes—exhibit female/male gender stereotypes to a disturbing extent (Bolukbasi et al. 2016). A seminal study on search engines showed how algorithms systematically retrieved racist and sexist search results, from keywords such as ‘black girl’, which was termed “algorithmic oppression” (Noble 2018). Yet, in comparison to the salient and influential research findings on unwanted bias relating to gender and race in AI systems, the category of age—critical to the study of social exclusion and social inequalities—has been largely neglected in existing research. Age bias in AI is only now starting to emerge as a critical problem requiring urgent action, in academic research (Chu et al. 2022; Rosales and Fernández-Ardèvol 2019) as well as in public policies. The World Health Organization (WHO) expressed concerns that, if left unchecked, AI technologies may perpetuate existing ageism in society and undermine the quality of health and social care that older people receive (WHO 2022). The scarcity of scientific investigation of age biases renders the topic of ageism in AI *terra incognita*.

The aim of this paper is to rectify this gap by pointing to several theoretical aspects and empirical illustrations of manifestations of ageism in AI. There is a plethora of ways AI had been defined, from simple formulations to complex definitions relating to the technological, scientific, and societal implications of the systems. This article follows the understanding of AI as a “socio-technical ecosystem,” which recognizes the interaction between people and technology and how complex infrastructures affect and are affected by society and by human behaviour (Dignum 2022). Machine learning can be defined broadly as a field at the intersection of statistics and computer science that uses algorithms to extract information and knowledge from data (Molina and Garip 2019) and, more specifically, a subfield of artificial intelligence that gives computers the ability to learn without explicitly being programmed (Brown 2021). This paper provides two main contributions to the debates on bias and fairness of AI, diversity, and inclusion in AI, as well as discussions on AI for social good. Firstly, it proposes a new analytical concept—*AI ageism*—with the aim of theoretically elucidating the various ways ageism can manifest in AI and creating a roadmap for identification of further critical areas in need of empirical research and policy intervention. Secondly, the paper provides illustrations and identifies areas where the application of AI technologies can become harmful or discriminatory to older populations.

Ethics of AI, which can be considered a critical theory (Waelen 2022), is an upcoming field of research that deals with the ethical assessment of emerging AI applications and addresses the new kinds of moral questions AI raises. The concepts of bias and fairness of AI belong to the wider ethical debate among academics, practitioners, and policymakers (Fjeld et al. 2020; Kordzadeh and Ghasemaghaei 2021; Mittelstadt et al. 2016; Tsamados et al. 2022), where algorithmic fairness appears to be a “wicked problem” with no clear agreement on problem statement or solution (Woodruff et al. 2018). The broad concept of fairness is rooted in philosophy, mathematics, ontology, sociology and law and can be applied for AI and ML mostly by the use of various fairness metrics (Wachter et al. 2020). Moreover, the concept of fairness is a situational, evolving, and highly contestable one and can only be understood in reference to the different social groups. Thus, the vast majority of algorithmic fairness frameworks are stipulated with reference to particular  social groups, often requiring a formal encoding of the groups into the dataset and/or algorithm (Hanna et al. 2020). Identification of vulnerable social groups is the way technical understanding of bias and fairness in ML (Wachter et al. 2021) is linked with the wider societal impact of AI and the variety of interdependencies between different actors in the realms of AI. The societal risk mitigation propositions such as “the society-in-the-loop” (Rahwan 2018), which combines the concept of “human-in-the-loop” control paradigm with mechanisms for negotiating the values of various stakeholders affected by AI systems or the concept of “representational harm” in ML (Barocas et al. 2017) reflect the concern of the AI community that the broad societal and marginalized group interests need to be integrated into the AI development processes. Furthermore, due to the lack of diversity among engineers and researchers in AI, and more broadly in digital technologies, the products that are developed and used by billions of users may result in the proliferation of bias on a large scale. Hence, inclusion and diversity in AI are crucial (Chou et al. 2018; Zhang et al. 2021). Sociologists, along with critical social scientists, can advance the conversation on bias, fairness, transparency, and accountability by transforming it into one of inequality, discrimination, power and hierarchy, and social exclusion, but also social good and social responsibility (Airoldi 2022; Zajko 2021).

## AI for social good and the ageing population

What is “social good”? At minimum, it is a conceptual framework that is filled with discursive content, as mentioned above, fairness, bias, or inclusivity, by various social and political actors. Different approaches to understanding the concept of the social good have been suggested. However, there is still only limited understanding about what constitutes AI for social good (also AI4SG) and different frameworks emerge with the intent to substantiate and advance the development and application of AI for social good. Generally speaking, AI for social good initiatives is successful to that degree they reduce, mitigate, or eradicate a given problem of moral weight. Accordingly, a working definition of AI for social good was coined: “the design, development, and deployment of AI systems in ways that (i) prevent, mitigate, or resolve problems adversely affecting human life and/or the wellbeing of the natural world, and/or enable socially preferable and/or environmentally sustainable developments” (Floridi et al. 2020, pp. 1773–1774). Against this background, applying artificial intelligence to humanitarian and environmental problems has become a broadly agreed upon coordination point in the discourse of “AI for social good”. The United Nations’ (UN) 17 Sustainable Development Goals (SDGs) has become a leading framework to manage the objectives of such efforts (Tomašev et al. 2020). Among ten guidelines to inform future AI4SG initiatives identified by an international collaboration of actors and stakeholders, the issues of fairness and inclusivity are included and reference to the category of age comes across: “Applications of AI need to be inclusive and accessible, and reviewed at every stage for ethics and human rights compliance (…) Unfairness may result in violations of the right to equality, manifesting as inequity in model performance and associated outcomes across race, ethnicity, age, gender, etc.” (Tomašev et al. 2020, p. 3). Altogether, the questions about the agenda of AI for social good are deeply intertwined with wider ethical and political issues regarding the legitimacy of decision-making with, and about, AI (Floridi et al. 2020).

Simultaneously, AI for social good is part of a growing movement^1^ that seeks to integrate ethics into AI to achieve more equilibrium between societal needs and technological progress. Previously remarkable attributions of data-driven systems, such as its disruptive potential, automation capabilities, and scaling of revenue are increasingly being codified as insufficient, even naïve, within the ethically aligned regime (Lee and Helgesson 2020). Some of the main debates within the movement concern problems such as algorithmic bias (Kordzadeh and Ghasemaghaei 2021), fairness vs accuracy (Aler et al. 2022), intricacies of keeping society in the loop (Rahwan 2018), and explaining results derived from black-boxed algorithms (Kitchin 2017). Within this framework, AI for social good implies that it should be rendered bias free and meet the principle of fairness and non-discrimination, in addition to bringing positive social impacts in the application of systems. For example, an algorithm is seen as satisfying the principle of fairness when the calculational outcomes are independent of sensitive variables, which indicates membership in vulnerable categories. The outcomes of those algorithms should be further examined for a non-discriminatory impact on salient social groups (Heinrichs 2022).

Furthermore, the ‘social good’ is something which benefits the “general public, being ‘for’ the people, and at the same time, it is something which reflects and respects their wishes, being ‘from’ the people” (Trotta, Lomonaco, Ziosi, 2021). This framework focusses on the “receiver” or “user” side of technology and reflects the humanistic approach to regulate AI which puts humans at the centre of AI inspection. With regard to the category of older persons, the categorization ‘for’ people can be understood twofold: firstly, as AI systems serving the particular needs of older people (e.g., in the care sector, transportation, smart housing) the purpose of which is to facilitate, manage, and support the ageing processes of individuals and societies; secondly, it implies AI should be designed according to the principles of universal design—that is, a technology which responds to the needs of all age groups and does not prioritize or exclude any demographic fraction—an ageless AI. To further substantiate the principle of AI ‘from’ people, the focus needs to shift to the diversity of persons engaged in development, design, production, and implementation stages of AI technology. The inclusion of older populations as “relevant social groups” (Pinch and Bijker 1987) in the process of construction of technology is an inevitable step into safeguarding the fairness and inclusiveness of the products and services in the field of AI. Actors involved in the social process of construction of technology speak from different backgrounds, experiences, and capabilities—transforming our understanding of the social good into a reflection of a myriad of perspectives. Questions arise: Whose perspectives can be articulated? What articulated perspectives become sanctioned as a legitimate contribution to the discourse? Virginia Dignum (2021) recently noted that still many stakeholders are not invited to the table, not joining the conversation. I argue that the older population is one group and social category that not only is being excluded from processes of development and deployment of AI, but is also invisible in the debate on ethical, inclusive, and fair AI. For older persons, the concerns include, but are not limited to, discriminatory impacts in health care, housing, employment, banking, and finance issues (Orwat 2020). The dearth of research on age biases in AI might be one of the reasons the debates and approaches to fairness and ethics in AI have until now not explicitly recognized that age should be included in the catalogue of protected socio-demographic characteristics.

## Ageing population, ageism, and technology

The urgency to investigate ageism in AI stems not only from the technological acceleration, but primarily from the reality of demographic change, which defines the way societies are ageing around the globe. In Europe, for example, the current median age amounts to 43.9 years and is projected to increase to 48.2 years until 2050 (Eurostat 2021). WHO estimates that by the end of 2030, the number of people 60 years and older will grow by 56%, from 962 million (2017) to 1.4 billion (2030). This rapid increase created momentum for the UN to proclaim the Decade of Healthy Ageing (2021–2030), where combating ageism is seen as a fundamental strategy for securing healthy and dignified futures for older adults. Simultaneously, the World Health Organization launched a global campaign to fight ageism and issued its very first *Global Report on Ageism* (WHO 2021). Despite decades of research, implementation of anti-discrimination policies and legislation, ageism is still alive and well (Stypinska and Turek 2017) or even, as recent studies show, has dramatically intensified during the COVID-19 pandemic (Ayalon et al. 2021).

Age is a complex and critical category in the study of social inequalities and social exclusion. The meaning of age goes significantly beyond being a bare number. It is a socially constructed multi-layered concept including biological, psychological, social, and economic dimensions (Marshall and Katz 2016; Vincent 1995). Further, the process of ageing itself is highly diverse, individualized and context-dependent, which contributes to intensification of inequalities due to accumulation of disadvantages throughout the life course (Ferraro and Shippee 2009). Hence, older adults are an extremely heterogenous group with different needs, potentials, and capacities. Ageism, however, is the only prejudice which will inevitably affect everyone, regardless of their gender, race, or other characteristic. Despite its ubiquitous nature, it is still a type of discrimination, which is not recognized as easily as sexism or racism as it often operates in a more subtle, yet corrosive manner. Ageism manifests in all domains of public and private life, and takes on multiple forms and expressions (WHO 2021). Therefore, it should not come as a surprise that ageism has found its way to manifest in digital forms, and more precisely in the AI and ML systems and technologies (Chu et al. 2022) as recently acknowledged by the World Health Organization’s Policy Brief on Ageism in AI for health (WHO 2022).

The definition of ageism has shifted from its basic understanding as “a process of systematic stereotyping and discrimination against people because they are old” (Butler 1975) to elaborated and multidimensional conceptualizations reflecting the multifaceted nature of ageism “as negative or positive stereotypes, prejudice and/or discrimination against (or to the advantage of) elderly people on the basis of their chronological age or on the basis of a perception of them as being ‘old’ or ‘elderly’. Ageism can be implicit or explicit and can be expressed on a micro-, meso- or macro-level” (Iversen et al. 2009, p. 15). Theories of ageism are following the digitalization of the phenomenon, that is, the presence of age biases, stereotypes, prejudice, and discrimination in their digital form. The concept of “visual ageism” for example, responds to this change and describes” the digital media practices of visually underrepresenting older people or misrepresenting them in a prejudiced way” (Ivan et al. 2020). Yet, the existing conceptualizations are still too narrow to address the complexity of various ageism manifestations in AI systems, algorithms and automatic decision-making systems. Moreover, they do not capture the reality and nature of human and non-human interactions—key to the era of AI and algorithms.

Social scientists have studied extensively the way older adults use and interact with digital technology (Katz and Marshall 2018; Loos et al. 2020; Wanka and Gallistl 2018)  and the way gerontechnology can assist older adults in adapting to ageing processes (Klimczuk 2012). The newly emerged theoretical framework of “Socio-gerontechnology” (Peine et al. 2021) promises to provide a unique understanding of ageing and technology from a social sciences and humanities perspective and contributes to the development of new ontologies, methodologies, and theories. However, the current ageing research with regard to intelligent technologies has been limited to several themes: the use of social robots and other smart technologies to assist and support active ageing and ageing in place (Pedersen et al. 2018), use of digital data in smart mobility (Sourbati and Behrendt 2020), and the use of smart technologies (e.g., wearable devices) in tracking and quantifying ageing bodies (Katz and Marshall 2018). Yet, the theoretical reflection and empirical analysis on the potential negative impact of these intelligent technologies and algorithmic systems on older persons has only very recently emerged as a necessary research agenda for sociology of ageing and social gerontology (Chu et al. 2022), and a thorough and systematic empirical and theoretical programme for the investigation of the phenomenon of ageism in AI is yet to be created.

The understanding of digital inequalities and the main focus has been on the concept of the “digital divide” (Choi et al. 2020; Van Dijk and Hacker 2003) and its corresponding three levels (Lutz 2019). In essence, the strength of the digital divide is that it makes us attentive to the division between digital insiders and excluded groups—outsiders—in terms of technological access and digital skill. In fact, age is the most consistent predictor of basic internet access and use (Hargittai and Dobransky 2017). Empirical studies strongly suggest that the older population is a significant part of the group that is systematically excluded from the digital ecosystem due to low digital literacy, internalization of existing negative stereotypes about older people as technically inept, or due to lack of interest (Gallistl et al. 2020; Hargittai and Dobransky 2017; Köttl et al. 2021). But how well do these patterns of digital divide translate from the digital sphere to the realm of AI?

Lutz (2019) argues that research on the third-level digital divide should include digital traces, algorithmic surveillance, and data-based discrimination into its syllabus. Further, Gran et al. (2020) suggest that the “digital divide” is facing a new frontier: awareness of algorithms. The authors used cluster analysis to measure levels of “algorithmic awareness” in a representative sample of the Norwegian population. By algorithmic awareness the study understood being aware of the algorithms’ functions and impacts on platforms, in services and search engines. They found out that in total, 41% of respondents expressed no awareness of algorithms, whereas the age group of 60–70 scored 61%, and 70 + scored 74%. In comparison to younger cohorts, the age group of 30–39 scored 15% which is strongly indicative of the age inequality in algorithmic awareness. Their findings show that awareness of algorithms is stronger in younger groups, with older people scoring lowest. Accordingly, this evidence suggests the continuous exclusion of the older population on a group level—an algorithmic divide understood as an extension, or another level of digital divide. The effects of algorithmic divide are believed to threaten to take away the various political, social, economic, cultural, educational, and career opportunities provided by machine learning and artificial intelligence (Yu 2020).

## Going beyond “bias” in AI: *AI ageism* as a researcher’s roadmap

Even though conceived of as mathematical formulas, algorithms are neither neutral, fair, nor objective. They reproduce the assumptions and beliefs of those who decide about their design and deployment. The variety of ways in which biases are encoded refers to the ways the technology is designed, the data is encoded, and the way in which people and the wider society interact with each other as well as with the different systems in place (Willson 2017). In their seminal work, Friedman and Nissenbaum (1996) identified three types of bias in computer systems: pre-existing from social institutions, technically created, and emerging bias from the context of use. Furthermore, the domination of AI ethics and fairness research by computer and data scientists is reflected in the use of language. The concept of “bias” originating from computer sciences dominates the scholarly discourses about exclusion in AI (Zajko 2021) and poses a risk of reducing the complexity of social inequalities to solely a technical level. Additional risks are presented by the substantial power asymmetries between those with the resources to design and deploy AI systems, and those who are classified, ranked, and assessed by these systems (Whittaker et al. 2019).

Therefore, by introducing the concept of *AI ageism*, I propose to go beyond the usage of “bias” as the dominant epistemological tool for understanding the negative effects of algorithmic models and systems. This concept proposes a broader socio-technical inquiry of different forms of ageism existing in AI understood as a socio-technical ecosystem (Dignum 2022). The working definition of *AI ageism*, open to empirical and theoretical refinement, conveys the following: practices and ideologies operating within the field of AI which exclude, discriminate, or neglect the interests, experiences, and needs of older populations and have or might have disparate impacts on age equality. It includes, but is not limited to, five interrelated forms: (1) age biases incorporated in algorithms and digital datasets (technical level), (2) age stereotypes, prejudices, and ideologies of actors in the field of AI (personal/actor level), (3) invisibility or clichéd representations of category of age and old age in discourses around AI (discourse level), (4) discriminatory effects of use of AI technology on older age groups (group level), (5) exclusion as users of AI technology, services and products (user level). This guiding analytical framework is aimed at delineating lines of research on different manifestations of ageism in AI by focusing on the forms which might be verified through empirical research and serve as an orientation framework for future social study of age and age inequalities in their algorithmic expression. The concept of *AI ageism* can facilitate the discovery of new data-driven manifestations of ageism and enhance the research by going beyond the limiting concept of “bias” (the technical level), which until now has strongly dominated the discourse and research about social fairness and equality of AI (Zajko 2021). The proposed analytical framework reflects on the need for understanding bias in algorithms as a multidisciplinary task (Ntoutsi et al. 2020), where it is acknowledged that roots of the problem are not only technological. Although bias in its technical form (level 1) is central to the definition of *AI ageism*, its full interpretation needs to be complemented by the other forms (2–5), which recognize the complex socio-technical interdependencies of the process of AI creation. Age stereotypes and prejudices are deeply ingrained in the social fabric, in interpersonal relations (2), as well as in discourses, images, and ideologies in the tech industry (3), and these manifestations of *AI ageism* need research attention to the same extent as the technical bias. This framework acknowledges the divisions on the continuum of the development of AI (from producer to end user), but proposes to go beyond the simple “end-user” perspective as too individualistic and introduces the “group level” as another dimension to which researchers should be particularly sensitive. The attention to this form of ageism—as a discrimination on the group level—reflects the concern about the impact of automating decision-making systems (ADMs), which do not require an active “end-user”, but where decisions are taken on behalf of them, often completely beyond their knowledge and awareness (Barocas et al. 2017; Eubanks 2018). Although the interdependencies between the forms of *AI ageism* can be identified analytically (e.g., the relation between the age stereotypes and prejudices of actors and the exclusion of older persons as users due to flawed design), their robustness needs to be tested empirically in a systematic way in the future.

## AI ageism in practice: illustrations

The identified dimensions of *AI ageism* are separated conceptually; however, there are significant interrelations and synergies between them. The proposed working definition should serve as an organizing tool for exploratory purposes, both on empirical and theoretical levels. The illustrations of the five dimensions of *AI ageism* provided in this section are based on scientific literature review, grey literature, and own preliminary research and observations. Hence, they are not exhaustive in the scope of the problem or the depth of investigation. Their sometimes fragmentary character reflects the unsystematic and incomplete character of research on ageism in AI.

### Age bias in algorithms and digital datasets (technical level)

Single studies indicate that age bias can occur in machine learning models or big data approaches. Diaz and colleagues (2019) analysed the treatment of age-related terms across 15 sentiment analysis models and 10 widely used GloVe word embeddings. Sentiment analysis is often used to measure opinions in product reviews or financial markets, but they are also useful in analysing political opinions expressed on social media. In the case of age-related bias, automated methods of opinion polling may falsely report more negative attitudes towards political issues or financial investments regarding age-related concerns, such as Medicare and Social Security. The study showed evidence that sentiment analysis disclosed significant age biases: sentences with ‘young’ adjectives were 66% more likely to be scored positively than identical sentences with ‘old’ adjectives (Díaz et al. 2019). Moreover, it proved that various sentiment analysis methods impact bias in outcomes, particularly that tools validated against social media data exhibit increased bias. Another study found relevant differences in the outcomes of face recognition models for predicting age and gender from photographs (Meade et al. 2021). The researchers used convolutional neural net (CNN) which is an advanced deep learning technique to analyse visual imagery. The model was trained on photos of celebrities from IMDb and Wikipedia, where their pictures were matched with their age, as well as data for general public from UTKFace data set. The results showed that age estimation generally performed poorly on older age groups (60 +), an effect which was compounded by gender and race; the age estimation worked disappointingly on older women of colour. Recently, another study showed that, when evaluating systems for facial emotion recognition (FER) using various classification performance metrics, the state-of-the-art commercial systems performed the best when recognizing emotions in younger adults (aged 19–31), and worst for the oldest age group (61–80) (Kim et al. 2021). In a similar vein, Korean researchers confirmed age, gender, and racial biases, including the intersectional bias, in popular face recognition models by using the face embedding association test (FEAT) to measure the biased way specific groups are being associated with particular attributes (such as pleasant/unpleasant, likeable/unlikable) (Lee et al. 2022).

The use of these ML systems and technologies is becoming widespread in everyday life and the age biases identified in ML models can have a direct or indirect impact on the ageing populations. It can be speculated that the effects on older persons will be predominantly tangible in the areas where for example face recognition systems will inadequately identify the age of the person or the identity of the person due to changes in facial biometric image due to age. Biometric technology has the potential to impact older persons more directly due to the way biological ageing impacts bodily functions. Touch, imaging, speech, and body language will all be impacted due to ageing processes. For instance, risk can relate to the age-linked fading away of fingerprints impacting the accuracy of their recognition (Rosales and Fernández-Ardèvol 2020).

In the case of face recognition, the major application where age is explicitly deployed, resides in the realm of age estimation techniques. The age estimation algorithms working on visual data require large datasets for training. Since an algorithm is only as good as the data it works with (Barocas and Selbst 2018), this is where ageism in digital datasets becomes apparent. Study of ageism in big data approaches confirms that the most ageist practices in intelligent systems design are related to data set limitations of the representativeness of the studied population and particularly to recruitment procedures that tend to exclude older people (Rosales and Fernández-Ardèvol 2019; Sourbati and Behrendt 2020). Most of the datasets include radical age cutoffs for their data. For example, The Face and Gesture Recognition Research Network (FG-NET) ageing database contains on average 12 pictures for each of its 82 subjects in varying ages between 0 and 69 years. Other datasets limit the ages at 70 (Tufts-Face-Database), 77 (MORPH), and the list continues. There are some outstanding exceptions to the rule, such as UTKFace dataset, where the photos depict adults up to the age of 116 years. The cutoffs are also visible in training data used for proprietary algorithms developed in the expanding sector of AI industry. For instance, the company YOTI configured its training data set with age brackets of 13–60 years and the highest estimated errors in performance of their algorithm are seen for the age group of 50–60 years. In its White Paper the company admits that, “it seems reasonable to hypothesise that any error will tend to be higher for older people than younger people, because older people will have been exposed to various unpredictable environmental factors for longer” (YOTI 2020). However, it is not only the age cutoffs that render the data infrastructures problematic for older populations. The human labelling processes and the classification of data into categories are also highly problematic, as images can be tagged in stereotyping and even offensive ways. In her intensive investigation of the ImageNet database—one of the most powerful visual data infrastructures—Kate Crawford demonstrates that the classifications for human images are, regardless of the supposed neutrality of any particular category, gendered, racialized, ableist, and ageist (Crawford and Paglen 2019).

### Stereotypes, prejudices, and ageist ideologies in the tech industry (personal/actor level)

The second identified dimension of AI ageism refers to the individual level of ageism among actors in the tech industry. Tech culture is homogenous in terms of age, ethnicity, and gender. It is young, predominantly populated by men of Caucasian or Asian origin, which is associated with the structural discrimination embedded in digital technologies (Wachter-Boettcher 2017). Ageism in the IT sector and tech industry is a well-known fact (Cook 2020; Marshall 2011; Rosales and Svensson 2021). In fact, Gullette notes “Silicon Valley can in fact be the most ageist place on the Earth” (Gullette 2017). Silicon Valley is the US centre for innovative technology companies and home to 2000 tech companies, the densest concentration in the world. Even more importantly, most of these companies are also leaders in their industries. These include software, social media, and other uses of the Internet, as well as AI. Silicon Valley sets standards for other firms. Companies around the world look to the tech giants to incorporate the same business models and management styles (Galloway 2018). Yet, these companies show rampant signs of various types of systemic biases and prejudice (Cook 2020; Park and Pellow 2004; Shih 2006; Wynn 2020), ageism being one of them. Large tech companies have phased out older workers over the past few years and continue to discriminate against anyone old enough to remember the 1980s. In 2007, a then 22-year-old Mark Zuckerberg famously admitted that tech companies should not hire people over 30 years because “Young people are just smarter”. Surveys carried out among tech workers only confirm that those blatant ageist statements are in fact the reality for the workers in Silicon Valley. A survey among American tech worker*s* shows that 76% of respondents say ageism exists in tech globally, whereas 80%of those in their late 40s say they are concerned their age (and ageism attitudes) will affect their careers (Dice 2018).

Ageism in the tech sector is specific as it is targeted at persons at a much younger age than in other sectors of the economy, where ageism starts to be experienced by someone as young as 45 years of age (Harris et al. 2018). In fact, online survey among tech workers shows that one-fourth of respondents in their early 30s already regard age as a barrier in getting a new job (Dice 2018). Another study, carried out among UK tech workers, revealed that on average, across the wider workforce people said they first started to experience ageism at work at an average age of 41 years—while IT and tech workers say they first experienced this at an average age of 29 years (Sevilla 2019). Therefore, it is argued that ageism in AI is partially fuelled by age biases prevalent in the tech industry, which are visible both in recruitment and hiring practices, as well as ideologies and beliefs related to concepts such as innovation and progress of the tech industry (Stypińska, Rosales, Svensson, forthcoming). Hence, the concept and ideology of disruptions coming from innovation and business theory can clarify ageism in tech. A disruptive innovation, which AI technology certainly is, is an “innovation that creates a new market and value network and eventually disrupts an existing market and value network, displacing established market-leading firms, products, and alliances” (Rahman et al. 2017). Disruptions are what drives innovation, progress, and success in Silicon Valley. These are the stories of “unicorn-startups”^2^ with implausible success or established tech giants that started out as the hobby of two geeks in their early 20s in a garage, which hold the collective imagination and frame the way success is understood. Those stories create an ideology that renders anyone over 30 years as incapable of innovation. Since AI research and development is at the forefront of technological innovation globally, it is plausible to assume these ideologies impact the way AI developers, software engineers, data scientists, and other AI practitioners work, think, and solve problems. In fact, studies on ageism in digital platforms indicate that the homophily of the community of software developers, who are predominantly young men of high socio-economic status, contributes to baking the prejudices and biases into the algorithms (Rosales and Fernández-Ardèvol 2020).

### Ageism in discourses about AI  (discourse level)

Studying discourses about algorithmic systems and processes is crucial to our understanding of the social power of algorithms and AI (Natale 2019; The Royal Society 2018). Next to the material power, algorithms can exert a discursive power revealing their political entanglements. The way algorithmic systems are spoken about is part of how they are fused into social and organizational structures and how they shape our imagination. Discourse is both constitutive and constituted where it simultaneously shapes, and is shaped by, social structures. Moreover, it is important to investigate how discourses about algorithms shape the broader debates about social change and development, and especially the role innovations play in the processes of development of AI (Beer 2017). By discourses on AI ethics, I refer here to the way the principles of AI ethics, such as fairness, bias, inclusivity, and diversity, are conveyed in documents and public debates with a particular focus on which social groups are iterated as those endangered by infringement of those principles.

The majority of initiatives and/or documents for inclusion and promotion of diversity in AI community are targeted at gender and racial minorities. Publications on bias in AI admit that “the most discussed forms of “unfair bias” in the literature relate to particular attributes or groups such as disabilities, race, gender, and sexual orientation” (Silberg and Manyika 2019). The most common formulations in the documents were, for example, “the diverse groups in terms of race, culture, gender, and socio-economic backgrounds”,^3^ or: “Hiring diverse backgrounds, disciplines, genders, races, and cultures”.^4^ The mainstream debates around the issues of AI fairness and inclusivity tend to omit the category of age and older persons. The absence can be observed in two forms: invisibility of old age as object of discussion and lack of representation of older persons as subjects in those discourses. This might be due to several factors, such as a relatively weak social representation of the rights of older persons in the area of AI, or ideologies and stereotypical beliefs about older persons as users or non-users of AI applications held by software producers.

Similar conclusions were drawn by a team of researchers at the University of Toronto who performed an analysis of documents listed in the repository of AI ethics guidelines created by Algorithm Watch, which as of April 2022, contains 173 documents created by governments, private entities, civil society, and international organizations. The search terms used in the analysis were “ageism” and similar notions like “age bias”, “age”, “old/older”, “senior(s)”, and “elderly”. The researchers found that in the 146 analysed documents that were available at that time, only 34 (23.3%) mention ageism as a bias for a total of 53 unique mentions. Out of these, 19 (54.7%) merely listed “age” as part of a general list of protected characteristics next to gender or race (Chu et al. 2022). The authors conclude that only 12 (8.2%) of the analysed documents provided somewhat more context about bias against older adults, but often no more than one or two sentences.

### Algorithmic discrimination–automatic decision-making (ADM) systems and their outcomes (group level)

This section highlights areas where deployment of AI can result in harm for older persons as a distinct demographic group. Two terms: “algorithmic discrimination” and “discrimination of algorithms” describe the negative *outcomes* of automated decision-making (ADM) systems or classification systems used in multiple AI applications (Kleinberg et al. 2018; Köchling and Wehner 2020; Orwat 2020). Discrimination occurs when the outcomes/outputs of ADMs infringe on the rights of persons based on their “protected” characteristic, such as gender, race, age, disability, or nationality (Orwat 2020). Although not all ADM systems are powered by AI, there is a stable increasing tendency towards more deployment of AI in those solutions (Chiusi et al. 2020b). Today, ADM systems intertwine with critical moments during a person’s life, for instance in shaping institutional access to higher education, insurance, financial services, and hiring decisions in the labour market (O’Neil 2016). For several years automatic decision-making systems have been under scrutiny for their opaque, erroneous, harmful or just false outcomes (Chiusi et al. 2020a, b; Noble 2018; O’Neil 2016). These systems can have the purpose of predicting, identifying, detecting, and targeting individuals or communities. ADMs are being increasingly used by private companies (e.g., in recruitment and personnel management) and public sectors (health care, education, social services, law enforcement) (Mittelstadt et al. 2016; Orwat 2020; Reisman et al. 2018).

With regard to ageing populations, the risk of discrimination lies in the way the biased algorithms are being used in practice in those realms of social, economic and cultural life where they could infringe on the rights of older persons. Their increased use in recruitment and hiring practices can threaten the way discrimination cases will be possible to detect (Köchling and Wehner 2020). Age discrimination in employment is one of the most wide spread types of discrimination in the labour market (Stypinska and Turek 2017) and the attempts to fight it with anti-discrimination legislation are challenging. An investigation by *ProPublica* revealed that Facebook ads can be and are targeted at precise age groups allowing employers to recruit job applicants that are below a certain age. The category of age can easily be used to create Facebook’s “affinity groups”, used to narrow or refine audiences, which are then used for targeting job advertisements to pre-selected candidates (Ajunwa 2019). Moreover, the already famous case of Amazon’s hiring algorithm downgrading the resumes of women gives a hint of what it could mean for older workers. For example, if a company tended to hire candidates who graduated from school (or landed their first job) by a certain date, it might introduce a bias towards younger candidates. The company’s software developers would need to actively monitor the system to ensure that something like that was not happening.

Another example where ADM could negatively impact large groups of older adults is the banking sector. In fact, more than the other ‘protected attributes’, age has the potential to affect credit access in a selective fashion, reducing it for some segments of society, while remaining benign for others. If a mortgage lending model found that older individuals have a higher likelihood of defaulting, it might reduce the lending options based on age leading to excluding older adults from those services (Silberg and Manyika 2019). The potential bias that such algorithms may generate against certain groups of people has also been increasingly acknowledged. In fact, in the proposed AI regulation of EU (2021), AI-systems used for credit scoring are designed as ‘high-risk’ and subjected to stringent regulations, which also necessitates further research in this area to collect empirical evidence of how these systems affect older demographic groups. The financial sector needs to be singled out as critical for investigation, since the digital exclusion of older adults has already raised serious concerns and attention. A campaign and a petition signed by more than 600,000 people called "I may be old, but I'm not an idiot" started by a Spaniard Carlos San Juan to stop exclusion of older people by banks emerged as a loud voice of those left behind by rapid digitalization processes (Müller 2022).

Documentation of severe social and personal consequences for individuals wronged by the outputs of such systems has raised questions about their fairness and even legality (Richardson 2019). A discussion is necessary which kind of ADM systems need to be assessed to what depth, depending on the potential damage for individual and society as a whole (Zweig et al. 2018). There should be a systematic assessment of the way in which older populations might be impacted by the increasing deployment of those systems in the private and public sector.

### Marginalization and exclusion of older persons as users (user level)

The last form of *AI ageism* discussed shortly is the exclusion as users. The compounded effects of ever-increasing complexity of digital technology and the already mentioned low algorithmic awareness among older adults (Gran et al. 2020) create structures which marginalize or exclude older persons as end users of AI technology. Ageism in technology design is not a new phenomenon. Studies exhibit different patterns in use of digital technology by older adults (Barbosa Neves and Vetere 2019; Gallistl et al. 2020). They show that older adults have very heterogenous patterns in use and “non-use” of the Internet (Gallistl et al. 2020); that older people are prone to self-stereotypes and self-exclusion in use of digital technology (Köttl et al. 2021); how older adults use smart watches and augmented reality games (Schlomann et al. 2019; Seifert 2020). Research suggests that older adults are generally portrayed as frail when described as users of AI assistive technology (Burema 2021). However, the question arises whether the data-driven technologies using AI and ML technologies pose any additional risks of harm and ageism?

An example of AI technology where older adults might experience ageism as users is the group of products called “conversational AI” which includes virtual assistants and chatbots. Conversational AI agents are increasingly used by companies for customer services and by consumers as personal assistants. The most famous examples of personal virtual assistants are probably Apple’s Siri, Amazon’s Alexa or Microsoft’s Cortana. Chatbots, used predominantly in customer service, are software applications used to conduct an online conversation via text. This technology is not scripted by humans and responds to human interlocutors using learning and human-guided algorithms (Schiebinger et al. 2011–2020). The challenge is that unless corrected for, the virtual assistant also learns and replicates human biases in the dataset (Schlesinger et al. 2018). Recent studies showed that virtual assistants and chatbots can exhibit racism and sexism (Schiebinger et al. 2011–2020; Cave and Dihal 2020). Similar problems might occur when testing virtual assistants and chatbots for their sensitivity to issues of age and ageism. Claims are being made that chatbots and virtual assistant are already ageist and sexist in the way they are profiled (usually as young women), but the question is whether they could also be ageist in their conduct (e.g., treat older customers unfairly or exhibit ageist stereotypes—jokes, etc.). Furthermore, if assistants implemented in a health care application perform more poorly with seniors, it could impact the quality of care provision and ultimately the health of the user. Further examples of areas where older adults might experience ageism as users of AI-driven technology include diverse smartphone applications where incomplete data for older age cohorts might result in inaccurate results (Rosales and Fernández-Ardèvol 2020). Further research is needed to reveal other aspects of this form of *AI ageism*.

## Conclusions

The aim of this paper is to turn scholarly attention to ageing populations as a socio-demographic group that can be defined as vulnerable, in relation to data-driven social transformations resulting from increasing use of AI technology in all realms of modern life. By introducing a new concept of *AI ageism*, this article contributes to the scholarly efforts to advance our knowledge of the harmful ways AI can impact the vulnerable group of older adults. The working definition of *AI ageism* with its five interrelated forms aspires to embody the complex and multifaceted character of ageism in the realm of AI. Furthermore, I argue that it is essential to go beyond the understanding of inequalities in AI dictated by the narrow use of the term “bias”. As social scientists, we are aware of the structural, institutional, and otherwise “non-quantifiable” forms of injustice and oppression in our social world (Wachter et al. 2020; Zajko 2021).

The increase in datafication, the advancements of AI such as deep learning and proliferation of operative AI in society, and the lack of knowledge of ageism in AI mutually reinforce the urgency of knowing how the category of age relates to AI and how principles of AI for social good could be implemented here. The COVID-19 pandemic accelerated the processes of digitalization and datafication. Since the beginning of this health crisis, data and advanced digital technologies have played a central role in how we respond and adapt to this situation. As a result, ethical principles such as trust, transparency, accountability, and privacy have been put to the test on a global stage. The current debates on ethical AI happening globally at all levels of stakeholders, from public entities, through large and small companies, AI practitioners and scholars from various disciplines show the urgency of the need to regulate AI with regard to its ethical standing. The landscape of currently drafted regulations, recommendations and guidelines for ethical AI is voluminous and diverse (Hagendorff 2020). Concurrently, in the “2021 Pew Research Centre Report”, experts expressed doubt that ethical AI design will be broadly adopted as the norm within the next decade, pointing to several challenges of such an ambitious endeavour, including (among others): the relational character of ethics, the importance of specific context of applying AI, the proprietary, hidden and complex nature of most AI design, the obstacles in governance of ethical AI, as well as the nature and relative power of the actors involved in any given scenario (Rainie et al. 2021). It is particularly the relatively low power of representation of ageing populations in the many phases of AI development, as well as their invisibility in the discourses and debates on ethical AI that requires our consideration.

Attempts to create more inclusive, diverse, and fair AI are necessary, even if flawed, inconsistent, or potentially unimplementable, as they have the potential to raise public awareness to these intricate issues. A large portion of the older population is unfamiliar with the complexity of AI, algorithms, or big data (Gran et al. 2020) and also do not want to engage with this new technology due to a lack of trust in these developments. To enable informed decisions on their part, communicative efforts must be made to explain various aspects of AI in formats that older age groups can respond to. The theoretical frameworks, as well as the emerging social movement captured under “AI for social good”, thus qualify as such an attempt and open a space for shaping the ways in which AI is and will be used in society. In particular frameworks emphasizing the humanistic approach to AI, such as AI for People, have great potential to include the perspectives of the vulnerable, underrepresented, and precarious social groups and include them in the participatory schemes of AI design. Victoria Dignum, an AI ethicist, notes “the elephant in the room is the huge blind spot we all have about our own blind spots. We correct bias for the bias we are aware of. An inclusive, participatory, approach to design and development of AI systems will facilitate a wider scope” (Dignum 2021, p.7).

The research on bias in AI has gathered significant momentum. In June 2021, the European Commission Research Program, Horizon Europe, issued a call for proposals to study bias in AI. However, in alignment with the argumentation made in this article, two shortcomings can be observed. Firstly, this call refers primarily to gender and race discrimination, although allows for incorporation of further biases. Secondly, it favours research on technical aspects of the biases with little mention of the socio-cultural or political implications of research on AI biases. Undoubtedly, the research on bias in AI needs to go beyond these two limitations and include not only age and ageing populations as relevant categories for researching digital inequalities, but also pay attention to the way these inequalities can be compounded in an intersectional way. Demographic change and the increasing proportion of older people in the population structure will have grave implications for the way digital and data-driven technologies will be used. Similarly, AI has the potential to transform the way we age and experience old age. Future research and efforts to design ethical AI should bring attention to synergies between these two megatrends and avoid operating in a vacuum or with a limited vision of future.

## Footnotes

1 Including the work of such organizations or platforms as: Algorithmic Justice League, AI for People, or Algorithm Watch.

2 The term „Unicorn-Startups” refers to those companies with a valuation more than $1 billion.

3 From: “The Toronto Declaration: Protecting the right to equality and non-discrimination in machine learning systems”.

4 From “In pursuit of inclusive AI”, publication of Microsoft.

## References

[CR1] Airoldi M (2022). Machine habitus towards a sociology of algorithms.

[CR2] Aizenberg E, van den Hoven J (2020). Designing for human rights in AI. Big Data Soc.

[CR3] Ajunwa I (2019). Age discrimination by platforms. Berkeley J Employ Labor Law.

[CR4] Aler A, Flavia T, Gökhan R, Julian K, Mendez A (2022). Ethical implications of fairness interventions: what might be hidden behind engineering choices ?. Ethics Inf Technol.

[CR5] Ayalon L (2021). Aging in times of the COVID-19 pandemic avoiding ageism and fostering intergenerational solidarity. J Gerontol Ser B Psychol Sci Soc Sci.

[CR6] Barbosa Neves B, Vetere F (2019). Ageing and emerging digital technologies designing and evaluating emerging technologies for older adults.

[CR7] Barocas S, Crawford K, Shapiro A, Wallach H. (2017) The problem with bias: allocative versus representational harms in machine learning. In SIGCIS Conference

[CR8] Barocas S, Selbst AD (2018). Big data’s disparate impact. SSRN Electron J.

[CR9] Beer D (2017). The social power of algorithms. Inf Commun Soc.

[CR10] Bolukbasi T, Chang K-WW, Zou J, Saligrama V, Kalai A (2016). Man is to computer programmer as woman is to homemaker? debiasing word embeddings. Adv Neural Inf Process Syst.

[CR11] Brown S. (2021) Machine learning, explained. MIT Sloan School of Management

[CR12] Buolamwini J, Gebru T (2018). Gender shades: intersectional accuracy disparities in commercial gender classificatio. Proceed Mach Learn Res.

[CR13] Burema D (2021). A critical analysis of the representations of older adults in the field of human–robot interaction. AI Soc.

[CR14] Butler R (1975). Why survive? Being old in America.

[CR15] Cave S, Dihal K (2020). The whiteness of AI. Philos Technol.

[CR16] Chiusi F, Fischer S, Spielkamp M (2020). Automated decision-making systems in the COVID-19 pandemic: a European perspective.

[CR17] Chiusi F, Fisher S, Kayser-Bril N, Spielkamp M (2020b) Automating society 2020. Algorithm Watch, Berlin. https://automatingsociety.algorithmwatch.org/

[CR18] Choi EY, Kim Y, Chipalo E, Lee HY, Meeks S (2020). Does perceived ageism widen the digital divide? And does it vary by gender?. Gerontologist.

[CR19] Chou J, Ibars R, Murillo O. (2018) In Pursuit of Inclusive AI. Microsoft Res. Microsoft

[CR20] Chu CH, Nyrup R, Leslie K, Shi J, Bianchi A, Lyn A (2022). Digital ageism: challenges and opportunities in artificial intelligence for older adults. Gerontologist.

[CR21] Commission E (2020). On artificial intelligence—a European approach to excellence and trust.

[CR22] Cook K (2020). Psychlogy of Silicon Valley.

[CR23] Crawford K, Paglen T. (2019) Excavating AI: the politics of images in machine learning training sets. https://excavating.ai/

[CR24] Cruz TM (2020). Perils of data-driven equity: safety-net care and big data’s elusive grasp on health inequality. Big Data Soc.

[CR25] Díaz M, Johnson I, Lazar A, Piper AM, Gergle D (2019). Addressing age-related bias in sentiment analysis. IJCAI Int Joint Conf Artif Intell.

[CR26] Dice (2018) Dice diversity and inclusion report. Available at: https://www.techhub.dice.com/2018-DI-Report-ResourceLibrary.html

[CR108] Dignum V (2021) The myth of complete AI-fairness. In: Tucker A, Henriques Abreu P, Cardoso J, Pereira Rodrigues P, Riaño D (eds) Artificial intelligence in medicine. AIME 2021. Lecture notes in computer science, vol 12721. Springer, Cham. 10.1007/978-3-030-77211-6_1

[CR27] Dignum V (2022) Relational artificial intelligence. 10.48550/arXiv.2202.07446

[CR28] Eubanks V (2018). Automating inequality: how high-tech tools profile, police, and punish the poor.

[CR109] Eurostat (2021) Baseline projections: demographic balances and indicators. Office for Official Publications of the European Communities, Luxembourg

[CR29] Ferraro KF, Shippee TP (2009). Aging and cumulative inequality: how does inequality get under the skin?. Gerontologist.

[CR30] Fjeld J, Achten N, Hilligoss H, Nagy A, Srikumar M (2020). Principled artificial intelligence: mapping consensus in ethical and rights-based approaches to principles for AI. Berkman Klein Center Internet Soc Res Publ Series.

[CR31] Floridi L, Cowls J, King TC, Taddeo M (2020). How to design ai for social good: seven essential factors. Sci Eng Ethics.

[CR110] Friedman B, Nissenbaum H (1996) Bias in computer systems. ACM Trans Inf Syst 14(3):330–347. 10.4324/9781315259697-23

[CR32] Gallistl V, Rohner R, Seifert A, Wanka A (2020). Configuring the older non-user: between research, policy and practice of digital exclusion. Social Incl.

[CR33] Galloway S (2018) The four: the hidden DNA of Amazon, Apple, Facebook, and Google. Portfolio

[CR34] Gran AB, Booth P, Bucher T (2020). To be or not to be algorithm aware: a question of a new digital divide?. Inf Commun Soc.

[CR35] Gullette MM (2017). Ending ageism, or how not to shoot old people, Ending Ageism, or How Not to Shoot Old
People.

[CR36] Hagendorff T (2020). The ethics of AI ethics: an evaluation of guidelines. Mind Mach.

[CR37] Hanna A, Denton E, Smart A, Smith-Loud J. (2020) Towards a critical race methodology in algorithmic fairness. FAT* 2020—Proceedings of the 2020 Conference on Fairness, Accountability, and Transparency, 501–512. doi:10.1145/3351095.3372826

[CR38] Hargittai E, Dobransky K (2017). Old dogs, new clicks: digital inequality in skills and uses among older adults. Can J Commun.

[CR39] Harris K, Krygsman S, Waschenko J, Laliberte Rudman D (2018). Ageism and the older worker: a scoping review. Gerontologist.

[CR40] Heinrichs B (2022). Discrimination in the age of artificial intelligence. AI & Soc.

[CR41] Ivan L, Loos E, Tudorie G (2020). Mitigating visual ageism in digital media: designing for dynamic diversity to enhance communication rights for senior citizens. Societies.

[CR42] Iversen TN, Larsen L, Solem PE (2009). A conceptual analysis of ageism. Nordic Psychol.

[CR43] Katz S, Marshall BL (2018). Tracked and fit: FitBits, brain games, and the quantified aging body. J Aging Studies.

[CR44] Kim E, Bryant D, Srikanth D, Howard A. (2021) Age bias in emotion detection: an analysis of facial emotion recognition performance on young, middle-aged, and older adults. AIES 2021—Proceedings of the 2021 AAAI/ACM Conference on AI, Ethics, and Society (Vol. 1). Association for Computing Machinery. doi:10.1145/3461702.3462609

[CR45] Kitchin R (2017). Thinking critically about and researching algorithms. Inf Commun Soc.

[CR46] Kleinberg J, Ludwig J, Mullainathan S, Sunstein CR (2018). Discrimination in the age of algorithms. J Legal Anal.

[CR47] Klimczuk A (2012). Supporting the development of gerontechnology as part of silver economy building. Ad Alta: J Interdisciplinary Res.

[CR48] Köchling A, Wehner MC (2020). Discriminated by an algorithm: a systematic review of discrimination and fairness by algorithmic decision-making in the context of HR recruitment and HR development. Bus Res.

[CR49] Kordzadeh N, Ghasemaghaei M (2021). Algorithmic bias: review, synthesis, and future research directions. Eur J Inf Syst.

[CR50] Köttl H, Gallistl V, Rohner R, Ayalon L (2021). But at the age of 85? Forget it!: internalized ageism, a barrier to technology use. J Aging Studies.

[CR51] Lee F, Helgesson CF (2020). Styles of valuation: algorithms and agency in high-throughput bioscience. Sci Technol Human Values.

[CR52] Lee S, Oh S, Kim M, Park E. (2022) Measuring embedded human-like biases in face recognition models †. In AAAIWorkshop on Artificial Intelligence with Biased or Scarce Data (AIBSD) (pp. 1–14). MDPI Comptuter sciences and mathematics forum

[CR53] Loos E, Sourbati M, Behrendt F (2020). The role of mobility digital ecosystems for age-friendly urban public transport: a narrative literature review. Int J Environ Res Public Health.

[CR54] Lutz C (2019). Digital inequalities in the age of artificial intelligence and big data. Human Behav Emerg Technologies.

[CR55] Marshall VW (2011). A life course perspective on information technology work. J Appl Gerontol.

[CR56] Marshall BL, Katz S (2016). How old am I?. Digital Culture Soc.

[CR57] Meade R et al (2021) Bias in machine learning: how facial recognition models show signs of racism, sexism and ageism. https://towardsdatascience.com/bias-in-machine-learning-how-facial-recognitionmodels-show-signs-of-racism-sexism-and-ageism-32549e2c972d

[CR58] Mittelstadt BD, Allo P, Taddeo M, Wachter S, Floridi L (2016). The ethics of algorithms: mapping the debate. Big Data Soc.

[CR59] Molina M, Garip F (2019). Machine learning for sociology. Ann Rev Sociol.

[CR60] Müller S (2022) Spanien: Carlos San Juan Gegen die Banken DW. Available online at: https://www.dw.com/de/spanien-carlos-san-juan-gegen-die-banken/a-60685233

[CR61] Natale S (2019). If software is narrative: Joseph Weizenbaum, artificial intelligence and the biographies of ELIZA. New Media Soc.

[CR62] Noble SU (2018). Algorithms of oppression: how search engines reinforce racism.

[CR63] Ntoutsi E, Fafalios P, Gadiraju U, Iosifidis V, Nejdl W, Vidal M-E (2020). Bias in data-driven artificial intelligence systems-an introductory survey. Wires Data Mining Knowl Discov.

[CR64] O’Neil C (2016). Weapons of math destruction: how big data increases inequality and threatens democracy.

[CR65] Orwat C (2020). Risks of discrimination through the use of algorithms.

[CR66] Park LSH, Pellow DN (2004). Racial formation, environmental racism, and the emergence of Silicon Valley. Ethnicities.

[CR67] Pedersen I, Reid S, Aspevig K (2018). Developing social robots for aging populations: a literature review of recent academic sources. Sociol Compass.

[CR68] Peine A, Marshall BL, Martin W, Neven L (2021). Socio-gerontechnology interdisciplinary critical studies of ageing and technology.

[CR69] Pinch TJ, Bijker WE, Bijker WE, Hughes TP, Pinch TJ (1987). The social contruction of facts and arftifacts: or how the sociology of science and the sociology of technology mights benefit each other. The social construction of technological systems.

[CR70] Rahwan I (2018). Society-in-the-loop: programming the algorithmic social contract. Ethics Inf Technol.

[CR111] Rahman AA, Hamid U, Chin T (2017) Emerging technologies with disruptive effects. Perintis E J 7(2):308–314

[CR71] Rainie L, Anderson J, Vogels E. (2021) Experts doubt ethical AI design will be broadly adopted as the norm within the next decade. Pew Research Center

[CR72] Reisman D (2018). Algorithmic impact assessments: a practical framework for public agency
accountability.

[CR73] Richardson R (2019). Confronting black boxes: a shadow report of the New York City automated decision system task force.

[CR74] Rosales A, Fernández-Ardèvol M (2019). Structural ageism in big data approaches. Nordicom Rev.

[CR75] Rosales A, Fernández-Ardèvol M (2020). Ageism in the era of digital platforms. Convergence.

[CR76] Rosales A, Svensson J (2021). Perceptions of age in contemporary tech. Nordicom Rev.

[CR77] Schlesinger A, O’Hara KP, Taylor AS (2018). Let’s talk about race: identity, chatbots, and AI. Conf Human Factors Computing Syst—Proceed.

[CR78] Schlomann A, Rasche P, Seifert A, Schäfer K, Wille M, Bröhl C, Geroimenko V (2019). Augmented reality games for health promotion in old age. Augmented reality games II: The gamification of education, medicine and art.

[CR112] Schiebinger L, Klinge I, Sánchez de Madariaga I, Paik HY, Schraudner M, Stefanick M (eds) (2011–2021). Gendered Innovations in Science, Health & Medicine, Engineering and Environment

[CR79] Seifert A, Gao Q, Zhou J (2020). Smartwatch use among older adults: findings from two large surveys. Human aspects of IT for the aged population technologies, design and user experience.

[CR80] Sevilla C (2019) Everyday ageism in tech industry. CW JOBS. https://www.cwjobs.co.uk/advice/ageism-in-tech

[CR81] Shih J (2006). Circumventing discrimination: gender and ethnic strategies in silicon valley. Gend Soc.

[CR82] Silberg J, Manyika J. (2019) Tackling bias in artificial intelligence (and in humans) McKinsey. Notes from the AI frontier: Tackling bias in AI (and in humans), 1–8

[CR83] Sourbati M, Behrendt F (2020). Smart mobility, age and data justice. New Media Soc.

[CR84] Stypinska J, Turek K (2017). Hard and soft age discrimination: the dual nature of workplace discrimination. Eur J Ageing.

[CR85] The Royal Society. (2018) Portrayals and perceptions of AI and why they matter. London

[CR86] Tomašev N, Cornebise J, Hutter F, Mohamed S, Picciariello A, Connelly B (2020). AI for social good: unlocking the opportunity for positive impact. Nat Commun.

[CR87] Trotta A, Lomonaco V, Ziosi M. (2021) Call for papers for special issue on AI for People. AI and Society

[CR88] Tsamados A, Aggarwal N, Cowls J, Morley J, Roberts H, Taddeo M, Floridi L (2022). The ethics of algorithms: key problems and solutions. AI & Soc.

[CR89] Tufekci Z (2014) Big questions for social media big data: representativeness, validity and other methodological pitfalls. Proceedings of the Eight International AAAI Conference on Weblogs and Social Media, pp 505–514. 10.1016/0022-5193(78)90170-4

[CR90] Van Dijk J, Hacker K (2003). The digital divide as a complex and dynamic phenomenon. Information Soc.

[CR91] Vincent J (1995). Inequality and old age.

[CR92] Wachter S, Mittelstadt B, Russell C (2020). Why fairness cannot be automated: Bridging the gap between eu nondiscrimination law and ai. arXiv.

[CR93] Wachter S, Mittelstadt B, Russell C (2021) Bias preservation in machine learning: the legality of fairness metrics under EU non-discrimination law. West Virginia Law Review, Vol 123, issue 3, pp 1–51

[CR94] Wachter-Boettcher S (2017). Technically wrong: Sexist apps, biased algorithms, and other threats of toxic tech.

[CR95] Waelen R (2022). Why AI ethics is a critical theory. Philos Technol.

[CR96] Wanka A, Gallistl V (2018). Doing age in a digitized world—a material praxeology of aging with technology. Front Sociol.

[CR97] Whittaker M, Alper M, Bennett CL (2019). Disability, Bias, and AI.

[CR98] WHO. (2021) Global report on ageism. Geneva

[CR99] WHO. (2022) Ageism in AI for health: WHO Policy Brief. Geneva

[CR100] Willson M (2017). Algorithms (and the) everyday. Inf Commun Soc.

[CR101] Woodruff A, Fox SE, Rousso-Schindler S, Warshaw J (2018). A qualitative exploration of perceptions of algorithmic fairness. Conf Human Factors Computing Sys—Proceed.

[CR102] Wynn AT (2020). Pathways toward change: ideologies and gender equality in a silicon valley technology company. Gend Soc.

[CR103] YOTI. (2020) White Paper Yoti Age Scan—Public version

[CR104] Yu PK (2020). Legal studies research paper series the algorithmic divide and equality in the age of artificial intelligence. Florida Law Rev.

[CR105] Zajko M (2021). Conservative AI and social inequality: conceptualizing alternatives to bias through social theory. AI Soc.

[CR106] Zhang D et al (2021) AI Index Report. Stanford University HAI. https://aiindex.stanford.edu/ai-index-report-2021/

[CR107] Zweig KA, Wenzelburger G, Krafft TD (2018). On Chances and risks of security related algorithmic decision making systems. Eur J Security Res.

